# The CRISPR/Cas9 System for Targeted Genome Engineering in Free-Living Fungi: Advances and Opportunities for Lichenized Fungi

**DOI:** 10.3389/fmicb.2019.00062

**Published:** 2019-02-07

**Authors:** Karthik Shanmugam, Sivaprakash Ramalingam, Gayathri Venkataraman, G. N. Hariharan

**Affiliations:** ^1^M.S. Swaminathan Research Foundation, Chennai, India; ^2^Department of Mycology, University of Bayreuth, Bayreuth, Germany; ^3^CSIR-Institute of Genomics and Integrative Biology, New Delhi, India

**Keywords:** free-living fungi, lichenized fungi, TALENS, CRISPR/Cas9, ZFNs, SSNs, mycobiont culture, transcriptome analysis

## Abstract

Studies using whole genome sequencing, computational and gene expression, targeted genome engineering techniques for generating site-specific sequence alterations through non-homologous end joining (NHEJ) by genomic double-strand break (DSB) repair pathway with high precision, resulting in gene inactivation have elucidated the complexity of gene expression, and metabolic pathways in fungi. These tools and the data generated are crucial for precise generation of fungal products such as enzymes, secondary metabolites, antibiotics etc. Artificially engineered molecular scissors, zinc finger nucleases (ZFNs), Transcriptional activator-like effector nucleases (TALENs; that use protein motifs for DNA sequence recognition in the genome) and CRISPR associated protein 9 (Cas9;CRISPR/Cas9) system (RNA-DNA recognition) are being used in achieving targeted genome modifications for modifying traits in free-living fungal systems. Here, we discuss the recent research breakthroughs and developments which utilize CRISPR/Cas9 in the metabolic engineering of free-living fungi for the biosynthesis of secondary metabolites, enzyme production, antibiotics and to develop resistance against post-harvest browning of edible mushrooms and fungal pathogenesis. We also discuss the potential and advantages of using targeted genome engineering in lichenized fungal (mycobiont) cultures to enhance their growth and secondary metabolite production *in vitro* can be complemented by other molecular approaches.

## Introduction

Fungal products are important in agriculture, textile, food, pharmaceutical industries, (Cowan, 2001; Chang and Miles, 2004) and in bioremediation. In nature, fungi play an important role in nutrient cycling, such as decomposition of dead organic matter (Molina et al., 1993; Keizer, 1998; Pilz and Molina, 2001) and act as biofertilizers (Hunt, 1999; Gates et al., 2005). Recent advances in fungal molecular biology have enabled us to sequence complete genomes, and carry out gene and protein expression arrays which further provide opportunities for development of novel products such as mammalian proteins, antibiotics, pigments, and fatty acids for human welfare using fungi (Demain, 1999; Adrio and Demain, 2003; Idnurm and Meyer, 2014). The selection of potential fungal strains for protein and metabolite biosynthesis is made on the basis of production yields and regulatory concerns, especially for fungi used in the pharmaceuticals and food industry. Several species of fungi are currently being used for large-scale biosynthesis of recombinant proteins and metabolites (Punt, 2006). Geographically, tropical forests are considered as “treasure-boxes” for fungal biotechnologists due to high levels of fungal biodiversity (Hawksworth and Kalin-Arroyo, 1995). Approximately 20% of all known fungal species and ~50% of ascomycetes are obligate symbionts in lichens (fungi that live in symbiotic association with algae or cyanobacteria (Hawksworth and Hill, 1984). Metabolites produced by fungi in particular, are well-known for their therapeutic potential; but a vast resource of these metabolites are unexplored and their potential needs to be tapped. Lichens have been long neglected by mycologists and overlooked by the industry for its relevant novel secondary compounds, its derivatives, proteins and genetic material for the development of useful products. Lichen compounds belong to aliphatic, cyclo-aliphatic, aromatic, and terpenic compound classes and are structurally unique and pharmaceutically relevant (Müller, 2001). Lichen metabolites have been screened for numerous biological activities viz. antiviral, antibiotic, antitumor, allergenic, plant growth inhibitory, anti-herbivore, and enzyme inhibitory properties (Boustie and Grube, 2005). Some compounds were found to have immense potential. The lichen compound diploicin isolated from *Buellia canescens*, served as a base to derive anilinoaposafranine, an important drug against *Mycobacterium tuberculosis* (Barry, 1969). Usnic acid, a dibenzofuran isolated from several lichen species was used as a major ingredient in many antibiotics, cosmetics, and sunscreen products (Oran et al., 2016). However, in nature lichens are slow growing, low biomass producing, and also crust forming, making large scale harvest difficult (Balaji et al., 2006). In recent years, mycobiont and whole thallus cultures of several lichen species were established for the biosynthesis of secondary compounds (Valarmathi and Hariharan, 2007; Kinoshita et al., 2015; Molina et al., 2015; Muthukumar et al., 2016; Shanmugam et al., 2016). Cutting edge technologies such as transcriptomics and CRISPR can be used to elucidate gene networks contributing to slow growth rates of mycobionts in culture.

## CRISPR/Cas9 System in Bacteria, Archaea and Eukaryotes

**Figure 1:**
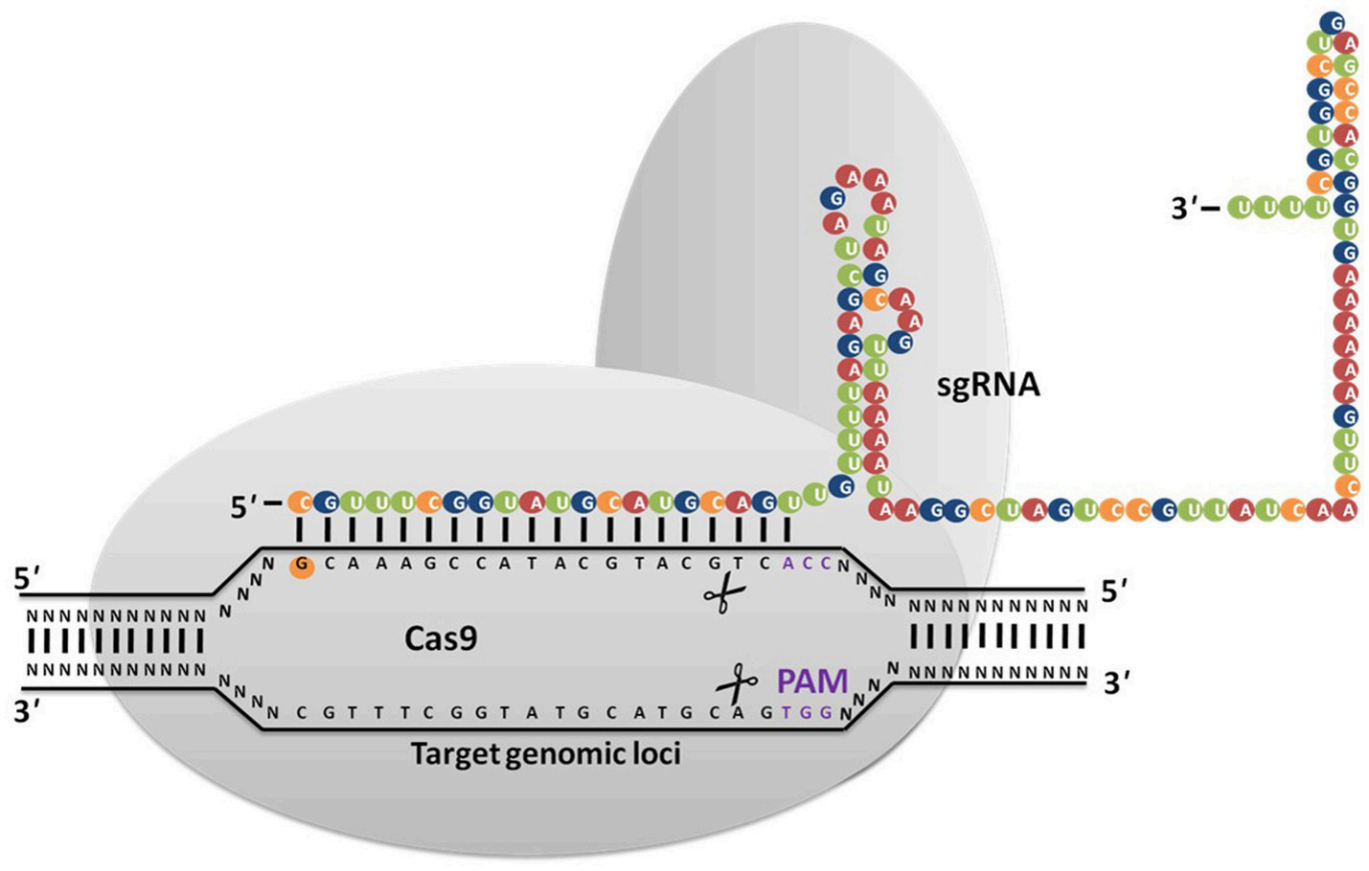
CRISPR/Cas9 system is based on RNA guided engineered nucleases that use complementary base pairing to recognize DNA sequences at target sites. Cas9 protein with nuclease activity induces DSB at the target site via direction of synthetic guide RNA (sgRNA) to the PAM sequence (NGG).

Viruses/phages infect both bacterial and archeal communities that have developed many strategies to evade infection as well as prevent virus adsorption to fend off viral DNA insertion (Horvath and Barrangou, 2010). In 1987, Nakata et al. (1987) discovered curious repeat and non-repeat sequences in the iap gene (Ishino et al., 1987) that are now referred to as Clustered Regular Interspaced Short Palindromic Repeats (CRISPR) (Jansen et al., 2002). Mojica et al. (2005) reported that these sequences consist DNA from bacteriophages that were shown to play a role in conferring adaptive bacterial immunity that prevents foreign DNA from being inserted into their genome as well as targets the invasive DNA for destruction. Bolotin et al. (2005) detected the existence of several Cas (CRISPR-associated proteins) genes, which encode DNA endonucleases, suggesting that foreign DNA degradation may be the primary function of CRISPR/Cas9. Three types of CRISPR systems such as Type I, II, and III were identified in 2011, of which the Type II CRISPR system has revolutionized genome editing in eukaryotic cells because of its relative simplicity (Mali et al., 2013). In 2012, Feng Zhang and his co-workers demonstrated its applicability for genome editing in prokaryotes and eukaryotes (Jinek et al., 2012; Cong et al., 2013; Jiang et al., 2013; Doudna and Charpentier, 2014; Nissim et al., 2014; Sander and Joung, 2014). The CRISPR/Cas9 system (Figure 1) is made up of three basic components: CRISPR RNAs (crRNAs), transactivating crRNA (tracrRNA), and CRISPR associated protein (Cas) (Ishino et al., 1987). A single component called synthetic guide RNAs (sgRNA) generated by crRNA and tracrRNA fusion is sufficient to accomplish genome editing in the presence of the Cas endonuclease. The CRIPSR/Cas9 system recognizes DNA through a RNA-DNA interaction between the target site and a CRISPR based synthetic guide RNA. Cas9 nuclease makes double-strand breaks (DSB) at the target site complementary/near complementary to the short 20-nucleotide gRNA present adjacent to the protospacer-adjacent motif (PAM) sequence. Both the 20-base-pair (bp) guide sequence on the gRNA and the (PAM) motif, “NGG” present downstream of the unique target sequence determine the specificity of Cas9-mediated DSB (Gasiunas et al., 2012; Jinek et al., 2012). Till date, several publications have reported on effective genetic modifications using CRISPR/Cas9 in several eukaryotic organisms including filamentous fungi (Table 1). The development of a CRISPR/Cas9 system for fungi was first reported in 2013, in *Saccharomyces cerevisiae* for disruption of the *ADE 2, CAN1.Y* genes (DiCarlo et al., 2013). After this study was published, gene editing technology was widely applied in several strains of yeast to improve their industrial production by several researchers (Bao et al., 2014; Horwitz et al., 2015; Mans et al., 2015; Stovicek et al., 2015; Fabre Jessop et al., 2016) *Schizosaccharomyces pombe* (Jake et al., 2015).

**Table 1:** Applications of programmable site-specific nucleases in free-living and higher fungi.

**Targeted fungal strains**	**Gene editing tool employed**	**Targeted genes or sites**	**Method of gene delivery**	**Promoter of sgRNA**	**Type of modification**	**Findings or achieved traits**	**References**
*Agaricus bisporus*	CRISPR/Cas9	Polyphenol oxidase gene (*PPO*)	Not mentioned	Not mentioned	Gene disruption	Inhibiting the synthesis of polyphenol oxidase (PPO), an enzyme that causes browning in Mushroom which leads increase in shelf life of edible mushroom.	Waltz, 2016
*Alternaria alternata*	CRISPR/Cas9	Melanin pathway genes, *pksA* and *brm2*	PEG mediated Protoplast transformation	*gpdA*	Gene inactivation	Gene inactivation in *A. alternata*. They established pyr4 as a selection marker and GFP for protein tagging.	Wenderoth et al., 2017
*Aspergillus fumigatus*	CRISPR/Cas9	Polyketide synthase gene (pksP)	Protoplast transformation	*SNR52*	Gene disruption	Inhibiting the synthesis of dihydroxynaphthalene (DHN), melanin and constitutive cas9 expression not deleterious to growth of *A. fumigatus*.	Fuller et al., 2015
*Aspergillus fumigatus*	CRISPR/Cas9	Trypacidin biosynthetic pathway gene	Protoplast transformation	*gpdA*	Base pair insertion	Trypacidin production enabled in non-producing strains of *A. fumigatus* by single nucleotide insertion.	Weber et al., 2017
*Aspergillus aculeatus* *luchuensis* *brasiliensi* *carbonarius* *nidulans* *A. niger*	CRISPR/Cas9	*yA* gene *albA* gene *pyrG* gene	Protoplast transformation	*gpdA*	Base pair deletion and insertion	Common CRISPR/Cas9 system established for six different fungal species.	Nødvig et al., 2015
*Aspergillus oryzae*	CRISPR/Cas9	*wA* gene *yA* gene *pyrG* gene	Protoplast transformation	U6	Gene disruption	Common CRISPR/Cas9 system established to achieve desired phenotypic variation.	Katayama et al., 2016
*Aspergillus oryzae*	PtFg TALEN	(ligase D) *lig D* gene	PEG mediated Protoplast transformation	*glaA142*	Gene disruption	Targeted mutations to generate strains with specific phenotypes, industrial applications and deletions reduced due to the application of PtFg TALEN.	Mizutani et al., 2016
*Candida albicans*	CRISPR/Cas9	*ENO1, ADE2* genes	Lithium acetate method	SNR52	Gene disruption	Creation of strains with mutations in multiple genes, gene families, and genes that encode essential functions.	Vyas et al., 2015
*Candida glabrata*	CRISPR/Cas9	GPI-anchored aspartyl protease genes Serine/threonine kinase genes	—	pCYC1, *pRNAH1*	Gene disruption	Using *D. melanogaster* as a model of infection, they find that CRISPR-Cas9 is a suitable tool to engineer *C. glabrata*, disrupt any specific gene and studied its impact during the infection process.	Enkler et al., 2016
*Cryptococcus neoformans*	Class 2 CRISPR	*ADE2* gene	Biolistic particle delivery	*ACT1*	Gene disruption	Understanding its pathogenicity.	Arras et al., 2016
*Ganoderma species*	CRISPR/Cas9	*ura3* gene	PEG mediated transformation	*T7*	Gene disruption	CRISPR/Cas9 system with codon-optimized Cas9 and *in vitro* transcribed gRNA.	Qin et al., 2017
*Myceliophthora thermophila* *M. heterothallica*	CRISPR/Cas9	*cre-1, res-1, gh1-1, alp-1*	Agrobacterium mediated transformation	*U6*	Gene disruption	Increased celluase enzyme production.	Liu et al., 2017
*Neurospora crassa*	CRISPR/Cas9	*CLR-2* gene *GSY-1* gene	—	*β-tubulin*	Gene replacement	ß-tubulin promoter driven clr-2 strain shows increased expression of cellulases enzyme. Introduce a codon optimized Fire fly luciferase under the control of the gsy-1 promoter at the csr-1 locus.	Matsu-ura et al., 2015
*Penicillium chrysogenum*	CRISPR/Cas9-RNP	*PKS* gene 17	Protoplast transformation	*U6*	Gene disruption	Inhibit synthesis of green pigment formation in spores.	Pohl et al., 2016
*Phytophthora sojae*	CRISPR/Cas9	*RXLR* gene *Avr4/6* replaced with *NPT II* gene	PEG mediated Protoplast transformation	*RPL41*	Gene replacement	Control of this pathogen by reducing the pathogenecity of fungus.	Fang and Tyler, 2016
*Pyricularia oryzae*	PtFg TALENs	*SRS2* gene	PEG mediated Protoplast transformation	–	Gene replacement	Mutations in SRS2 result in a hyper-recombination phenotype, sensitivity to DNA damaging agents and synthetic lethality with mutation that affects DNA metabolism.	Arazoe et al., 2015
*Saccharomyces cerevisiae* *Kluyveromyces lactis*	CRISPR/Cas9	Resistant alleles to elevated temperature ethanol concentrations	Lithium acetate	*SNR52*	Gene disruption	Multiplex integration of point mutations Enhancement of ethanol resistance and integration of muconic acid pathway.	Horwitz et al., 2015
*Saccharomyces cerevisiae*	CRISPR/Cas9	*ADE2* gene *CAN1.Y* gene	Lithium acetate	*SNR52*	Gene disruption	Controlling purine biosynthetic pathway. More gRNAs can be easily synthesized and Combinatorial transformed. This could allow for genomic targeting of many loci at time.	DiCarlo et al., 2013
*Saccharomyces cerevisiae*	CRISPR/Cas9	*ADE2* gene *PDC1* and *PDC5* genes	PEG/LiAc method	*SNR52*	Gene disruption	Using the ADE2 gene as the disruption target and GFP as insertion fragment for simultaneous disruption and knock-in. Single step construction and alleles of PDC1 and PDC5 genes are disrupted and heterologous lactate dehydrogenase genes are inserted.	Stovicek et al., 2015
*Saccharomyces cerevisiae*	HI-CRISPR/Cas9	*CAN1, ADE2* and *LYP1* genes *ATF2, GCY1* and *YPR1* genes	LiAc/SS carrier DNA/PEG method	*SNR52*	Gene disruption	(HI-CRISPR) strategy represents a powerful tool for creating yeast strains with multiple gene 16 knockouts.	Bao et al., 2014
*Saccharomyces cerevisiae*	CRISPR/Cas9	Acetyl-CoA carboxylase Malonyl-CoA reductase gene *PDC1, ALD6, ACL1* and *ACL2* genes	LiAc/SS carrier DNA/PEG method	*TEF1*	Gene disruption	Acetyl-CoA production in the cytosol to increase 3-hydroxypropionic acid.	Fabre Jessop et al., 2016
*Saccharomyces cerevisiae*	CRISPR/Cas9	*ACS1, ACS2* genes replaced by pdhA, *pdhB, aceF, lpd, lplA*, and *lplA2*	LiAc/SS carrier DNA/PEG method	*SNR52*	Gene replacement	Achieved simultaneous integration of a multigene construct combined with gene deletion.	Mans et al., 2015
*Saccharomyces cerevisiae*	CRISPR/Cas9	*tHMG1* gene	Lithium acetate	*TEF1*	Gene disruption	Non-native β-carotene pathway was reconstructed in *S. cerevisiae* by simultaneous integration of three pathway genes into individual intergenic genomic sites	Ronda et al., 2015
*Saccharomyces cerevisiae*	CRISPRm	*NatR* gene nourseothricin-resistance	Lithium acetate	*RNR2*	Gene disruption	Metabolic engineering and strain improvement's	Ryan et al., 2014
*Schizosaccharomyces pombe*	rrk1CRISPR/Cas9	*rrk1* gene	Lithium Acetate/PEG/Heat-shock method	*rrk1*	Gene disruption	The rrk1 CRISPR-Cas9 method enables rapid and efficient genome manipulation and unlocks the CRISPR toolset for use in fission yeast.	Jake et al., 2015
*Talaromyces atroroseus*	CRISPR/Cas9	UA08_00425 encoding the naphtha-γ-pyrone *PKS* gene	PEG mediated Protoplast transformation	*gpdA*	Gene disruption	Explore the secondary metabolism of this fungus and identified a novel gene encoding a hybrid PKS-NRPS, which is responsible for production of medically relevant ZG-1494α.	Nielsen et al., 2017
*Ustilago maydis*	CRISPR/Cas9	*bE1*,*bW2* genes	Protoplast transformation	*U6*	Gene disruption	Controlling the pathogenecity of *Ustilago maydis*.	Schuster et al., 2016

## Development of CRISPR/Cas9 System for Filamentous Fungi and Higher Fungi

Similarly CRISPR/Cas9 technology was used to edit the genomes of various filamentous fungi and higher fungi including Ascomycetes and Basidiomycetes and fungus-like eukaryotic microorganisms. Programmable Site-Specific Nucleases for Targeted Genome Engineering in these fungi groups encouragingly have shown promising outcomes (Table 1). For example, the CRISPR/Cas9 system has now been applied and established in several fungal species such as *Agaricus bisporus* (Waltz, 2016); *Aspergillus* species (Nødvig et al., 2015); *Myceliophthora* (Liu et al., 2017); *Neurospora crassa* (Matsu-ura et al., 2015), *Phytophthora sojae* (Fang and Tyler, 2016); *Talaromyces atroroseus* (Nielsen et al., 2017); *Trichoderma reesei* (Liu et al., 2015), and *Ustilago maydis* (Schuster et al., 2016).

## Applications of Targeted Genome Engineering in Free-living Fungi

In filamentous fungi, substantial homology lengths (~1 Kb) with near 100% homology are required to achieve efficient homologous replacement leading to low recombinational efficiencies (HR; Weld et al., 2006). One strategy to circumvent this would be to increase either the expression of genes involved in promoting HR or decrease expression of genes involved in non-homologous end joining (NHEJ). In *Penicillium chrysogenum*, knocking out a gene conferring NHEJ (*hfdA*) resulted in efficient disruption of three genes related to beta-lactam antibiotic biosynthesis (Kück and Hoff, 2010). There was a significant increase in the osmotic sensitivity of the strain, suggesting that there is always some tradeoff that is incurred by disrupting the natural NHEJ process and disruption of NHEJ-related genes, and deficiency in NHEJ activity may promote genomic instability (Zhang and Jasin, 2011). In this background, using gene-editing nucleases that can bring about targeted genome engineering is an alternative advancement to the above-mentioned method and can prove to be a more precise and efficient method for targeted gene modifications in a free-living fungal system. Gene-editing nucleases can be used for experimental exploration of fungal gene function and enhancement of essential traits in economically important fungal species such as biosynthesis of secondary compounds, antibiotics, enzyme production, and increasing shelf life time of edible mushrooms.

Recent advancements in genome sequencing technologies and establishment of transformation protocols for industrially important fungi has now paved the way for fungal biotechnologists to efficiently use engineered nucleases for establishing important free-living fungal species with industrially important desired traits. Gene-editing nucleases are powerful tools for site-specific mutagenesis utilizing either NHEJ mediated repair pathway or homology-directed repair involving transgene integration at a target locus in the fungal genome. The first successful example of targeted genome engineering in fungi using CRISPR/Cas9 system was established in *Saccharomyces cerevisiae* (DiCarlo et al., 2013).

## Genome Editing for Postharvest Browning of Edible Mushrooms

Rapid postharvest browning of white button mushrooms is a process leads to substantial economic losses. Browning is initiated by enzymatic oxidation of phenols such as tyrosine and non-enzymatic phenol oxidation induced by different factors, including bacteria (Nerya et al., 2006). Knocking out a single polyphenol oxidase gene (PPO) in *A. bisporus* using CRISPR based deletion of specific bases in PPO results in a 30% reduction in PPO activity and concomitant reduction in browning. This leads to increased shelf life time of edible mushroom.

## Genome Editing for Secondary Metabolite, Antibiotics and Enzyme Production

The potential of fungal secondary metabolism to provide secondary metabolites has until recently been impeded by the lack of knowledge in available genetic tools for most of the fungal species. However, the emergence of several Site-Specific Nucleases (SSN)—mediated genome engineering technology: TALENs/CRISPR/Cas9-based genome editing systems adapted for several genera of industrially important filamentous fungi species, has now opened doors for future efforts in discovery and biosynthesis of desired quantity of natural products such as secondary metabolites, antibiotics, and enzymes and engineering their biosynthetic pathways for industrial applications. Several studies have reported successful genetic modifications in several eukaryotic organisms including filamentous fungi. For instance, Liu et al. (2015) showed efficient genome editing in filamentous fungus *T. reesei* using CRISPR/Cas9 system. Furthermore, it was demonstrated that multiple genome modifications can be generated simultaneously in *T. reesei* using CRISPR/Cas9 system. In another study, the Cas9 and sgRNAs were introduced through *Agrobacterium* mediated transformation (Liu et al., 2015). Recently, Matsu-ura et al. (2015) have shown efficient gene replacement in a model filamentous fungus, *Neurospora crassa* using CRISPR/Cas9 system. The authors replaced the endogenous promoter of *clr-2* with beta-tubulin promoter and introduced codon-optimized fire fly luciferase gene under the control of *gsy-1* promoter at the clr-2 and csr-1 loci, respectively. The introduced beta-tubulin promoter driven *clr-2* strain shows increased expression of cellulase (Matsu-ura et al., 2015). Very recently, Liu et al. (2017) have successfully used the CRISPR/Cas9 system for increasing the production of cellulase in *Myceliopthora thermophila* and *M. heterothallica*. In this study, the genes *cre-1, res-1, gh1-1and alp-1* responsible for synthesizing cellulase were disrupted simultaneously leading to hyper cellulase producing strains. A Cas9-sgRNA RNP mixture targeting the *PKS17* gene in *P. chrysogenum* resulted in absence of green pigment inspores, giving white colonies and a visual screen to examine efficiency of CRISPR mediated gene disruption (Pohl et al., 2016).

In 2016, Jessop-Fabre et al. engineered *S. cerevisiae* to produce 3-hydroxypropionic acid (3-HP) by inserting genes for 3-HP production (ACC carboxylase and malonyl coA reductase) derived from *Chloroflexus aurantiacus* using CRISPR coupled with a newly developed EasyClone-Marker Free toolkit. Further upstream engineering to boost cytosolic acetyl-CoA production to feed into 3-HP production utilizing genes from diverse organisms (Enterococcus faecalis; Pyruvate dehydrogenase complex), *Salmonella enterica* (PDC1, ALD6 and ACS) and *Yarrowia lypotica* (ACL1/ACL2 citrate lyase subunits; citrate transporter gene) was also examined using CRISPR. Using this genome engineering vector suite, single and triple insertions are obtained with 90–100 and 60–70% targeting efficiency, respectively.

## Genome Editing of Human and Plant Fungal Pathogens

### Human Fungal Pathogens

Fungal pathogens kill more people per year worldwide than malaria or tuberculosis. It is estimated that around 1.6–2 million people are affected every year because of fungal pathogens (Denning and Bromley, 2015). Major human pathogenic fungi occur in the genera *Cryptococcus, Candida*, and *Aspergillus*, of which the genus *Aspergillus* causes between 38 and 80% of fungal disease-associated mortality across the world.

Vyas et al. (2015) have demonstrated development of an efficient codon optimized Cas9 system to target genes in drug-resistant clinical isolate strains of diploid Candida. This paves the way to examine mechanisms that render Candida resistant to antifungals using CRISPR technology.

The fungal pathogen *Candida glabrata* has become the second most common causative agent of candidiasis in the world and it is a major public health concern. Enkler et al. (2016) demonstrated CRISPR/Cas 9 disruption of *GPI*-anchored aspartyl protease and serine/threonine kinase in *C. glabrata* reduces infectivity in the model invertebrate organism *Drosophila melanogaster*. Thus, the CRISPR-Cas9 system is a powerful tool to assess the potential role of candidate genes in *C. glabrata* infectivity. Arras et al. (2016) have demonstrated functionality of the CRISPR construct in Cryptococcus *neoformans* by disruption of the *ADE2* gene. It was also established that Cas9 carrying *C. neoformans* strains do not affect progression of virulence in a murine inhalation model and is thus a powerful tool to explore mechanisms of *C. neoformans* pathogenicity.

### Plant Fungal Pathogens

Plant pathogenic fungi extensively damage global crop production, affecting an estimated 10% of harvested crops impacting food security. Platinum–Fungal TALENs (PtFg TALENs) and CRISPR/Cas9 have now made gene editing possible in fungi (Arazoe et al., 2015; Matsu-ura et al., 2015; Nødvig et al., 2015). PtFg TALENs harbor nonRVD variations at specific residues and have been applied in rice blast fungus *Pyricularia oryzae* to improve the efficiency of homologous recombination-mediated targeted gene replacement by up to 100% demonstrating its efficacy for basic and applied biology in filamentous fungi (Arazoe et al., 2015). Using CRISPR/Cas9, the effector Avr4/6 of the soybean pathogen *P. sojae* was efficiently knocked out and precisely replaced by the selectable marker nptII, uncovering additional roles for the corresponding R gene loci, *RPS4* and *RPS6* in conferring resistance that do not involve recognition through Avr4/6 (Fang and Tyler, 2016). The basidiomycete *U. maydis* is the causative agent of corn smut disease. The utility of the CRISPR/Cas9 system in this fungal pathogen was established by disrupting the homeodomain complex protein encoding genes bE1 and bW2 with a visual phenotypic screen (filamentous) to pick disruptants due to loss of filament formation (Schuster et al., 2016). Establishment of the utility of gene editing tools in plant pathogenic fungus-like eukaryotic microorganisms such *P. sojae, P. oryzae*, and *U. maydis* will encourage further refinements in the technology and provide plant breeders and pathologists an additional weapon in their fight against fungal pathogens.

## Opportunities to Utilize Targeted Genome Engineering in Lichenized Fungi

**Figure 2:**
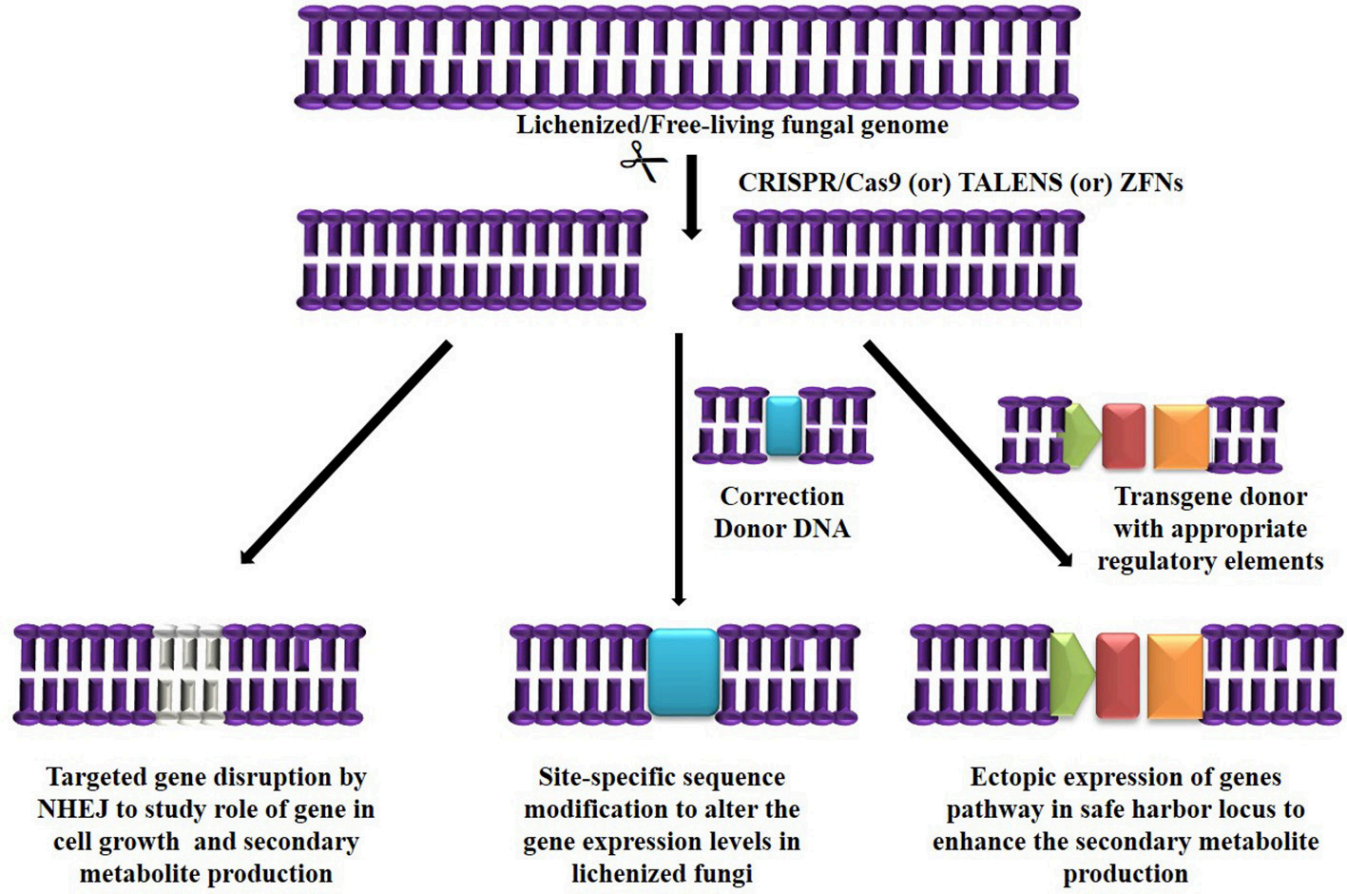
Targeted genome engineering in lichenized/free-living fungal genome using programmable SSNs.

**Figure 3:**
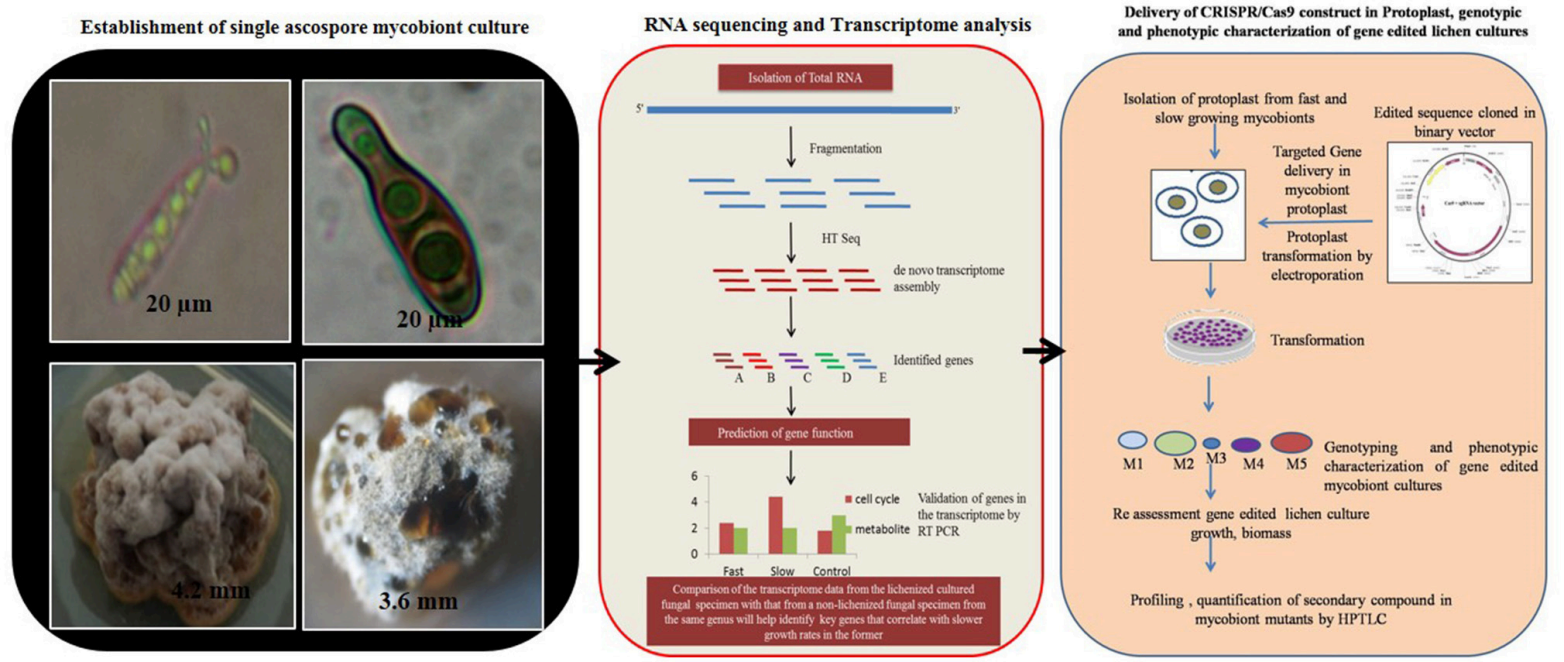
Hypothetical workflow of whole transcriptome and targeted genome engineering in ascospore derived lichen mycobiont culture.

The major challenge in culturing lichenized fungi are their slow growth rates in both natural and laboratory culture conditions. Molecular mechanisms contributing to slow growth rates need to be examined as they hinder establishment of mycobiont cultures. Unraveling these mechanisms will help in isolating known and novel bioactive secondary metabolites in desired industrial quantities using established *in vitro* systems. So far only few lichen forming fungal species have been sequenced for discovery of secondary metabolites and mechanisms conferring drought tolerance and symbiosis (Junttila and Rudd, 2012; Wang et al., 2014). However, there are no reports available regarding molecular mechanisms in lichenized fungi with special reference to factors such as growth inhibiting mechanisms, nutritional choices, key metabolic pathways and compound biosynthesis from the pure mycobiont cultures. Hence, we hypothesize (Figures 2, 3) that whole transcriptome analysis of cultured lichenized fungal species will reveal gene clusters associated with the above key factors. Comparison of the transcriptome data from the lichenized cultured fungal specimen with that from a free-living fungal specimen from the same genus will help identify key genes that correlate with slower growth rates in the former. Subsequently, functional validation of the predicted gene clusters can be achieved through targeted disruption of key genes using cutting-edge CRISPR/Cas9 technology to identify molecular mechanisms controlling growth rates. This will facilitate metabolic engineering of important genes to enhance growth potential of the lichenized mycobiont.

## Conclusions and Future perspective

Advancements in Next Generation Sequencing and targeted engineering of genomic regions using site specific nucleases has allowed more reliable targeted genome editing in eukaryotes including fungal genomes. In comparison to conventional genetic methods, CRISPR gene editing technology will provide more rapidly executable tools to carry out functional genomics in filamentous fungal species that target desired traits. This will need to be complemented by genomic (methylome) and small RNA profiling studies that could potentially silence gene clusters to related growth, development and biosynthesis of targeted natural products to realize the full potential of lichenized fungal metabolomics. This also includes exploring regions of the lichenized fungal genome.

## Author Contributions

KS, SR, and GH developed the concept and wrote the manuscript. KS and SR designed the schematic figures. GV contributed during revision and provided inputs to improvement the manuscript.

### Conflict of Interest Statement

The authors declare that the research was conducted in the absence of any commercial or financial relationships that could be construed as a potential conflict of interest.

## Abbreviations

CAZymes: Carbohydrate Active Enzymes
CRISPR: Clustered regularly interspaced short palindromic repeats
Cas: CRISPR associated proteins
NGS: Next generation sequencing
SSNs: Site specific nucleases
TALENs: Transcriptional activator-like effector nucleases
PAM: Protospacer associated motif
ZFNs: Zinc finger nucleases.
